# Establishment of nursing needs scale for elderly patients with urinary incontinence and its reliability and validity test

**DOI:** 10.3389/fmed.2025.1602675

**Published:** 2025-07-24

**Authors:** Xiangping Liu, Jing Pan, Silian Cai

**Affiliations:** ^1^Department of Gynaecology, Ruijin Hospital Affiliated to Shanghai Jiaotong University, Shanghai, China; ^2^Department of Respiratory Medicine, Ruijin Hospital Affiliated to Shanghai Jiaotong University, Shanghai, China

**Keywords:** elderly patients, urinary incontinence, nursing needs, scale development, reliability, validity, geriatric nursing, Delphi method

## Abstract

**Objective:**

Urinary incontinence significantly impacts the quality of life of elderly patients, yet there is a lack of specific assessment tools for their nursing needs in the Chinese healthcare context. To develop a nursing needs scale for elderly patients with urinary incontinence and test its reliability and validity, aiming to provide a quantitative assessment tool for geriatric nursing practitioners and a scientific basis for quality-of-life interventions and standardized nursing for these patients, thus promoting the standardization of nursing practice.

**Methods:**

A comprehensive approach was adopted, including literature analysis, qualitative interviews, the Delphi method, pre-testing, and questionnaire surveys. Literature was retrieved from multiple databases and relevant websites to construct the initial scale framework and item pool. Purposive sampling was used to select 12 elderly patients with urinary incontinence (aged 60 years and above) and 10 medical staff for semi-structured qualitative interviews. Twenty-two experts participated in two rounds of Delphi consultations. Convenience sampling was applied to select 30 patients for pre-testing and 530 patients for the formal questionnaire survey. Content validity was evaluated using the Item-Content Validity Index (I-CVI) and Scale-Content Validity Index (S-CVI). Kaiser-Meyer-Olkin (KMO) test and Bartlett’s test of sphericity were used to assess sampling adequacy. Factor loadings and total variance explained were calculated through exploratory factor analysis. Confirmatory factor analysis was conducted to validate the factor structure. The data were analyzed using SPSS 26.0 and AMOS 25.0 software to evaluate the scale’s reliability and validity.

**Results:**

The initial scale had 48 items in 5 dimensions. After expert consultations and item screening, the pre-test version with 36 items was formed. Through exploratory factor analysis on 250 patients, 5 common factors were extracted, and one item was deleted, resulting in the final scale. Confirmatory factor analysis on 280 patients showed that the model fit well (χ^2^/df = 1.412, RMSEA = 0.037, SRMR = 0.042, GFI = 0.901, TLI = 0.942, CFI = 0.947, NFI = 0.915, IFI = 0.948). The scale had good reliability (Cronbach’s *α* coefficient of the total scale was 0.901, split-half reliability was 0.865) and validity (content validity index S-CVI was 0.942).

**Conclusion:**

The developed nursing needs scale for elderly patients with urinary incontinence consists of 35 items in 5 dimensions. The scale demonstrates good psychometric properties and can serve as an effective assessment tool in clinical practice. However, further research with larger samples and different regions is needed to improve the scale.

## 1 Introduction

Urinary incontinence (UI) is a common yet often overlooked health problem among the elderly (1). It is defined as the involuntary leakage of urine through the urethra, which is closely related to factors like age-related pelvic floor muscle laxity and detrusor muscle degeneration (2, 3). The global prevalence of UI is considerable, with a large number of elderly people affected. In China, the reported incidence of UI in the elderly population ranges from 18.1–26%, mainly presenting as mild to moderate cases (3). Recent epidemiological studies have shown that UI prevalence increases with age, affecting up to 50% of women and 20% of men over 80 years old globally (4, 5). This condition has a multifaceted negative impact on the elderly, encompassing physical, psychological, and social aspects, and also brings a heavy economic burden (6).

The relevance of nursing care in UI management is grounded in the biopsychosocial model of health, which recognizes that effective UI management requires addressing not only physical symptoms but also psychological well-being and social participation. Within this theoretical framework, nursing care plays a crucial role in providing holistic support that encompasses skin care, psychological counseling, social reintegration, and family education.

In the Chinese context, the management of elderly patients with UI faces unique challenges. The healthcare coverage for UI patients varies significantly between urban and rural areas. In urban settings, patients typically have better access to specialized urological care and incontinence products through medical insurance reimbursement. However, in rural communities, access to professional assistance is limited, and many patients rely on family caregivers for daily management. The availability of incontinence products and specialized nursing services is also restricted in these areas, creating disparities in care quality (7).

Physically, UI can lead to various problems. The constant exposure of the skin to urine increases the risk of skin complications such as incontinence-associated dermatitis and pressure sores, especially for those with limited mobility (8, 9). UI restricts daily activities and social participation, leading to isolation (10). Nocturia, a particularly distressing symptom that significantly impacts quality of life in elderly UI patients, often leads to sleep disruption, increased fall risk, and daytime fatigue (11). Psychologically, UI often causes embarrassment and shame, which can lead to negative emotions like depression and anxiety (12). Studies show that a significant proportion of elderly UI patients experience such psychological issues, which further exacerbate the physical symptoms and form a vicious cycle (13).

Identifying and meeting the needs of elderly UI patients is crucial for improving their quality of life. By accurately assessing their needs, healthcare providers can develop personalized care plans. For instance, providing proper skin care can prevent skin problems, and offering pelvic floor muscle training can help improve urinary control (14). These targeted interventions can alleviate UI symptoms and enhance the overall well-being of patients.

However, currently, there is a lack of a specific and comprehensive assessment tool for the nursing needs of elderly UI patients. While existing general assessment tools for the elderly have been developed internationally, they present significant limitations when applied to UI patients. For example, the Barthel Index focuses primarily on basic activities of daily living without addressing UI-specific needs such as bladder training or incontinence product management. Similarly, the Comprehensive Geriatric Assessment (CGA) provides broad evaluation but lacks the specificity required for UI-related psychological support and social reintegration needs (15, 16). Furthermore, Western-developed tools often fail to account for Chinese cultural factors, such as the central role of family caregivers and traditional attitudes toward UI that may increase stigma and affect help-seeking behavior (17).

Therefore, this study aims to develop a nursing needs scale specifically designed for elderly UI patients within the Chinese healthcare context. Based on the Zuluaga-Raysmith (Z-R) model, which provides a comprehensive framework for assessing human needs across multiple dimensions (18), we seek to create a culturally appropriate and clinically relevant assessment tool. The scale will enable geriatric nursing practitioners to quantitatively evaluate patients’ nursing needs, contributing to more scientific and standardized care planning.

## 2 Methods

### 2.1 Research design

This study employed a methodological approach combining literature analysis, qualitative interviews, the Delphi method, pre-testing, and questionnaire surveys to develop and validate a nursing needs scale for elderly patients with urinary incontinence (19). The overall process adhered to strict scientific standards to ensure the reliability and validity of the scale. The study was conducted from January 31, 2024 to January 31, 2025, during the COVID-19 pandemic period, which may have influenced patient recruitment and representation.

### 2.2 Literature analysis

The literature analysis was conducted systematically to identify existing knowledge about nursing needs of elderly patients with urinary incontinence and relevant assessment tools. All retrieved literature was screened by two independent researchers using predefined inclusion and exclusion criteria.

The analysis process included: Data extraction: Key information was extracted from included studies, including study design, sample characteristics, assessment methods, nursing interventions, and patient outcomes.

Thematic analysis: Content was analyzed to identify recurring themes related to nursing needs across five main domains:

- Physical care needs (skin care, mobility assistance, hygiene management).
- Medical nursing needs (catheter care, medication management, complication prevention).
- Educational needs (self-management techniques, product usage, lifestyle modifications).
- Social participation needs (maintaining social activities, reducing isolation).
- Psychological support needs (coping strategies, emotional support, dignity preservation).

Gap identification: The analysis revealed a lack of comprehensive, culturally-specific assessment tools for elderly UI patients in the Chinese healthcare context, supporting the need for this scale development.

Framework development: Based on the literature findings and the Zuluaga-Raysmith (Z-R) model, an initial conceptual framework was constructed with 48 items across 5 dimensions to guide subsequent scale development.

### 2.3 Search strategy

A comprehensive search was conducted in eight domestic and foreign databases, including CNKI, Wanfang, PubMed, EMBASE, EBSCO, CINAHL, Web of Science, and The Cochrane Library. Additionally, relevant guideline websites such as Medlive Clinical Guidelines, NICE Guidelines, and the European Urology Association website were explored. The search terms included “elderly,” “urinary incontinence,” “nursing,” “treatment,” “needs,” “older people,” “urinary incontinence,” “care,” “treatment,” and “demand,” both in Chinese and English. The search covered the period from the establishment of each database to 2024, without language restrictions. A snowball-sampling approach was used, combining key terms and subject headings to retrieve relevant literature.

### 2.4 Inclusion and exclusion criteria

Inclusion Criteria: Literature related to the nursing of elderly patients with urinary incontinence, patients’ needs, diagnosis and treatment of urinary incontinence, rehabilitation management, and complications of urinary incontinence, as well as articles published in Chinese or English were included.

Exclusion Criteria: Conference abstracts, news reports, patents, and other literature without full-text access, as well as duplicate publications or those with incorrect data, were excluded.

### 2.5 Literature screening and data extraction

All retrieved literature was imported into Endnote X9 software to remove duplicates. Two independent researchers screened the non-duplicate literature based on the inclusion and exclusion criteria. In case of any disagreements, a third researcher was consulted until a consensus was reached. The relevant information from the included literature was then extracted to construct the initial scale framework and item pool.

## 3 Qualitative interviews

### 3.1 Selection of interviewees

Purposive sampling was employed to select 12 elderly patients with urinary incontinence and 10 medical staff (doctors and nurses) from two tertiary-level hospitals in ShangHai Province from January 31, 2024 to January 31, 2025. The sample size was determined based on the principle of data saturation, that is, until no new codes emerged. While our multidisciplinary expert panel included psychologists during the Delphi consultation phase, the qualitative interview phase focused on doctors and nurses as they represent the primary healthcare providers with direct daily contact with UI patients in the Chinese healthcare system. This approach allowed us to capture practical, frontline perspectives on nursing needs.

Inclusion Criteria for Elderly Patients with Urinary Incontinence: Age ≥ 60 years old, meeting the diagnostic criteria of the International Continence Society (ICS, defined as involuntary urine leakage through the urethra), and providing informed consent.

Exclusion Criteria for Elderly Patients with Urinary Incontinence: Patients with malignant tumors, genital tract bleeding, pelvic floor nerve denervation, mental illness, or cognitive impairment.

Inclusion Criteria for Medical Staff: Professionals with relevant qualifications (doctors and nurses), with at least 5 years of experience in the treatment and nursing of urinary incontinence, and providing informed consent.

Exclusion Criteria for Medical Staff: Those on external training, internships, or standardized training, and those absent from work for more than 3 months.

### 3.2 Development of interview outline

Semi-structured interview guides were developed based on the Health Belief Model and the biopsychosocial framework. An initial interview outline was developed based on the research objectives, literature review, and group discussions. Before the formal interviews, a simulated interview was conducted with a team member, followed by pre-interviews with two elderly patients with urinary incontinence and two medical staff (doctors and nurses). The final interview outline was then finalized.

Interview Outline for Elderly Patients with Urinary Incontinence: Questions covered the onset of urinary incontinence symptoms, self-perception of control, impact on life, treatment methods, difficulties in nursing, and additional support needed.

Interview Outline for Medical Staff: Topics included attention to patients’ needs, communication with patients about their needs, overlooked needs of elderly patients with urinary incontinence, and additional help required for patients’ recovery.

### 3.3 Data collection and quality control

Interviews were carried out in a quiet and private office by two researchers trained in qualitative research methods. Before the interviews, the researchers introduced themselves, explained the research purpose, and obtained informed consent from the interviewees. A combination of in-depth interviews and observation was used. The interview content was based on the interview outline, with questions asked in a progressive manner. Any unclear answers were promptly clarified, and the interview process was recorded using a Lenovo voice recorder for 30–60 min.

### 3.4 Data analysis

The data collected from the interviews were analyzed using Colaizzi’s seven-step method based on the theory of descriptive phenomenological qualitative research using NVivo 12 software. The steps included carefully reading the interview records, extracting meaningful statements, coding recurring viewpoints, classifying similar concepts, analyzing and describing the classified concepts, extracting theme concepts, and verifying the results with the interviewees. Representative themes and categories were selected to supplement and refine the scale item pool.

## 4 Delphi method

### 4.1 Selection of experts

A total of 22 experts from 17 different units in 7 cities across Shanghai and surrounding provinces were invited to participate in the Delphi consultation. The inclusion criteria for experts were as follows: working in the fields of urological nursing, nursing education, clinical psychology, or scale development methodology; having an intermediate professional title with at least 10 years of work experience in the field, or a senior professional title with at least 5 years of work experience; and being willing to participate actively in the study. The expert panel included 16 healthcare professionals (10 nurses, 4 urologists, 2 clinical psychologists), 4 nursing education researchers, and 2 methodology experts in scale development.

### 4.2 Development of expert consultation questionnaire

The expert consultation questionnaire was developed based on the preliminary scale dimensions and items. It consisted of three parts: a preface, an evaluation of the initial scale draft, and basic information of the experts. The evaluation of the scale draft included instructions for filling, item content, item importance, and scoring principles. The importance of scale items was rated using a Likert 5-level scale (1 = very unimportant, 2 = unimportant, 3 = moderately important, 4 = important, 5 = very important). Experts also self-evaluated their familiarity with the content, which included four aspects: practical experience (scored 0.5, 0.4, 0.3), theoretical knowledge (scored 0.3, 0.2, 0.1), reference to domestic and foreign materials (scored 0.1, 0.1, 0.05), and subjective perception (scored 0.1, 0.1, 0.05), with a total of five levels of familiarity (1.0, 0.8, 0.6, 0.4, 0.2).

### 4.3 Distribution and collection of questionnaires

Two rounds of expert consultations were conducted from March to May 2024. The questionnaires were distributed and collected by the research team members via email or WeChat Wenjuanxing. Experts were reminded to return the consultation results within 2 weeks. After the first round of consultation, the research team summarized and analyzed the expert opinions, and then conducted the second round of consultation after a 2-week interval. After the second round, the questionnaire items were further revised and improved until the experts’ opinions reached a relatively consistent level.

### 4.4 Item selection criteria

In the first round of expert consultation, items were selected if they met the following criteria simultaneously: mean importance score ≥ 3.50, coefficient of variation ≤ 0.25, and full-score ratio ≥ 40%. In the second round, items needed to meet the criteria of mean importance score ≥ 4.0 and coefficient of variation ≤ 0.20 to be selected.

## 5 Pre-test

### 5.1 Selection of research subjects

Convenience sampling was used to select 30 elderly patients with urinary incontinence from two tertiary-level hospitals in Shanghai. The inclusion and exclusion criteria were the same as those for the qualitative interviews.

### 5.2 Data collection and analysis

The pre-test version of the scale was administered to the selected patients. Their opinions on each item were collected, and through group discussions, items with ambiguous meanings were revised. This process aimed to improve the clarity and comprehensibility of the scale, forming the pre-test version of the scale.

## 6 Questionnaire survey

### 6.1 Selection of research subjects

For the formal questionnaire survey, 530 elderly patients with urinary incontinence from two tertiary-level hospitals in Shanghai were recruited using convenience sampling. The inclusion and exclusion criteria remained consistent with the previous stages. The collected data were randomly divided into two groups using a random number generator software. One group was used for exploratory factor analysis, and the other for confirmatory factor analysis.

### 6.2 Sample size calculation

For the test of surface validity, 20–30 participants were required, and 30 patients were finally included in the pre-test. For exploratory factor analysis, considering a sample-to-item ratio of 5–10 and a 10% invalid questionnaire rate, a minimum of 198 samples were needed. Eventually, 250 valid questionnaires were collected. We chose to split the data for EFA and CFA rather than conducting EFA on the full sample to avoid overfitting and to provide independent validation of the factor structure. This approach follows best practices in scale development methodology, ensuring that the confirmatory analysis is performed on a separate dataset from the exploratory analysis (20). For confirmatory factor analysis, a sample size of more than 200 or 5–20 times the number of items, and larger than the sample size of exploratory factor analysis was required. Finally, 280 valid questionnaires were obtained.

### 6.3 Research tools

General Information Collection Form: This form was developed by the research team based on literature review and clinical experience, as no validated instrument existed for collecting UI-specific demographic and clinical information in the Chinese population. This form collected information such as gender, age, educational level, marital status, income level, obesity, type of urinary incontinence, frequency of urine leakage, frequency of urination, number of childbirths, delivery method, constipation history, drinking water situation, gynecological or History of male surgery, number of diseases, and lifestyle habits affecting the disease.

Nursing Needs Scale for Elderly Patients with Urinary Incontinence: The five dimensions of the scale were derived from the Zuluaga-Raysmith (Z-R) model of human needs (18), which was adapted through our qualitative interviews and expert consultations to reflect the specific context of elderly UI patients in China. The scale consisted of 5 dimensions (daily care needs, medical nursing needs, health education needs, social participation needs, and psychological comfort needs) and initially had 36 items. Each item had 5 options: “not needed” (scored 1 point), “not too needed” (scored 2 points), “somewhat needed” (scored 3 points), “needed” (scored 4 points), and “very needed” (scored 5 points). The total score of the questionnaire ranged from 36 to 180 points, with higher scores indicating a higher degree of nursing care needs.

### 6.4 Data collection method

Trained investigators obtained the consent of the patients and distributed the questionnaires. A total of 68 patients (12.8%) required assistance in completing the questionnaire due to low educational level or physical limitations. Patients with a low educational level or those unable to complete the questionnaire independently were assisted by the investigators, who read the questions and recorded the patients’ answers. To minimize potential bias, investigators were trained to read questions neutrally without interpretation and to record responses verbatim. The questionnaires were collected and reviewed on the spot to ensure that there were no missing items, and each questionnaire took 15–20 min to complete.

### 6.5 Statistical analysis

Data were double-entered into EpiData 3.1 software and checked for errors to eliminate invalid questionnaires (21). SPSS 26.0 software was used for item analysis and exploratory factor analysis. Item analysis included the following methods: Correlation Coefficient Method: Items with a non-significant correlation coefficient (*p* > 0.05) and *r* < 0.4 were deleted.

Critical Ratio Method: The first 27% of the samples were defined as the low-score group, and the last 27% as the high-score group. An independent-sample t-test was used to compare the differences between the two groups. Items with a critical value (CR) < 3 or *p* > 0.05 were deleted.

Homogeneity Test: The Cronbach’s *α* coefficient after deleting each item was calculated, and items that caused a decrease in the coefficient were retained. Items that did not meet the above three criteria simultaneously were deleted.

Kaiser-Meyer-Olkin (KMO) measure and Bartlett’s test of sphericity were conducted to assess the suitability of data for factor analysis. KMO values > 0.9 indicate excellent sampling adequacy, values between 0.8–0.9 indicate good adequacy, and values > 0.6 are considered acceptable. Bartlett’s test with *p* < 0.05 indicates that correlations between items are sufficiently large for factor analysis (22).

For exploratory factor analysis, principal component analysis with varimax rotation was used. Factors with eigenvalues > 1 were retained, and items with factor loadings < 0.4 or cross-loadings (difference between highest and second-highest loading < 0.2) were considered for deletion. The total variance explained by the extracted factors should ideally exceed 60% (20).

Cronbach’s *α* coefficient, split-half reliability, and test–retest reliability were used to evaluate the reliability of the scale. Content validity and structural validity were used to evaluate the validity of the scale. Content validity included item-level content validity (I-CVI) and scale-level content validity (S-CVI). Exploratory factor analysis was performed using the principal component analysis method, and confirmatory factor analysis was carried out using the maximum-likelihood method in AMOS 25.0 software. The significance level was set at *α* = 0.05 for all statistical tests.

Additionally, to evaluate the criterion-related validity of the scale, we selected the Incontinence Quality of Life (I-QOL) instrument as an external criterion. The I-QOL is a widely used and validated tool for assessing quality of life in patients with urinary incontinence (23). Correlation analysis was performed between our scale and the I-QOL to determine the relationship between nursing needs and quality of life in elderly patients with urinary incontinence.

To assess the test–retest reliability, a subset of 50 participants were asked to complete the scale again after a two-week interval. The intraclass correlation coefficient (ICC) was calculated to evaluate the stability of the scale over time.

## 7 Results

A total of 22 interviewees were included, among whom 12 were elderly patients with urinary incontinence, denoted as P1-P12. Their ages ranged from 61 to 74 years old, with an average age of 67 years (SD = 4.2). There were 6 male and 6 female patients, 8 were married, 2 were divorced, and 2 were widowed. Five patients had overflow urinary incontinence, 5 had stress urinary incontinence, and 1 each had mixed urinary incontinence and urge urinary incontinence. The detailed information is shown in Table 1. The characteristics of our interview sample reflect recruitment patterns during the COVID-19 pandemic, which may have resulted in a younger cohort and underrepresentation of certain UI types.

**Table 1:** General information of elderly patients with urinary incontinence (*n* = 12).

Serial number	Gender	Age	Marital status	Type of urinary incontinence	Frequency of urine leakage	Number of pregnancies and deliveries	Mode of delivery
P1	Male	65	Married	Overflow Urinary Incontinence	2–3 times a week	None	None
P2	Male	63	Divorced	Overflow Urinary Incontinence	Approximately once a day	None	None
P3	Female	65	Married	Stress Urinary Incontinence	Approximately once a day	1 time	Vaginal Delivery
P4	Male	62	Married	Overflow Urinary Incontinence	2–3 times a week	None	None
P5	Female	70	Widowed	Stress Urinary Incontinence	Approximately once a day	2–3 times	Vaginal Delivery
P6	Female	66	Married	Stress Urinary Incontinence	2–3 times a week	2–3 times	Vaginal Delivery
P7	Male	61	Married	Overflow Urinary Incontinence	2–3 times a week	None	None
P8	Female	68	Married	Mixed Urinary Incontinence	Several times a day	≥3 times	Vaginal Delivery
P9	Female	65	Divorced	Stress Urinary Incontinence	2–3 times a week	2–3 times	Vaginal Delivery
P10	Male	74	Married	Overflow Urinary Incontinence	Approximately once a day	None	None
P11	Female	69	Widowed	Stress Urinary Incontinence	Once a week or less	2–3 times	Vaginal Delivery
P12	Male	73	Married	Urge Urinary Incontinence	Several times a day	None	None

Urinary incontinence types are defined as follows: Stress urinary incontinence—involuntary leakage on effort or exertion; Urge urinary incontinence—involuntary leakage accompanied by urgency; mixed urinary incontinence—combination of stress and urge symptoms; Overflow urinary incontinence—involuntary leakage associated with bladder overdistension.

**Figure 1:**
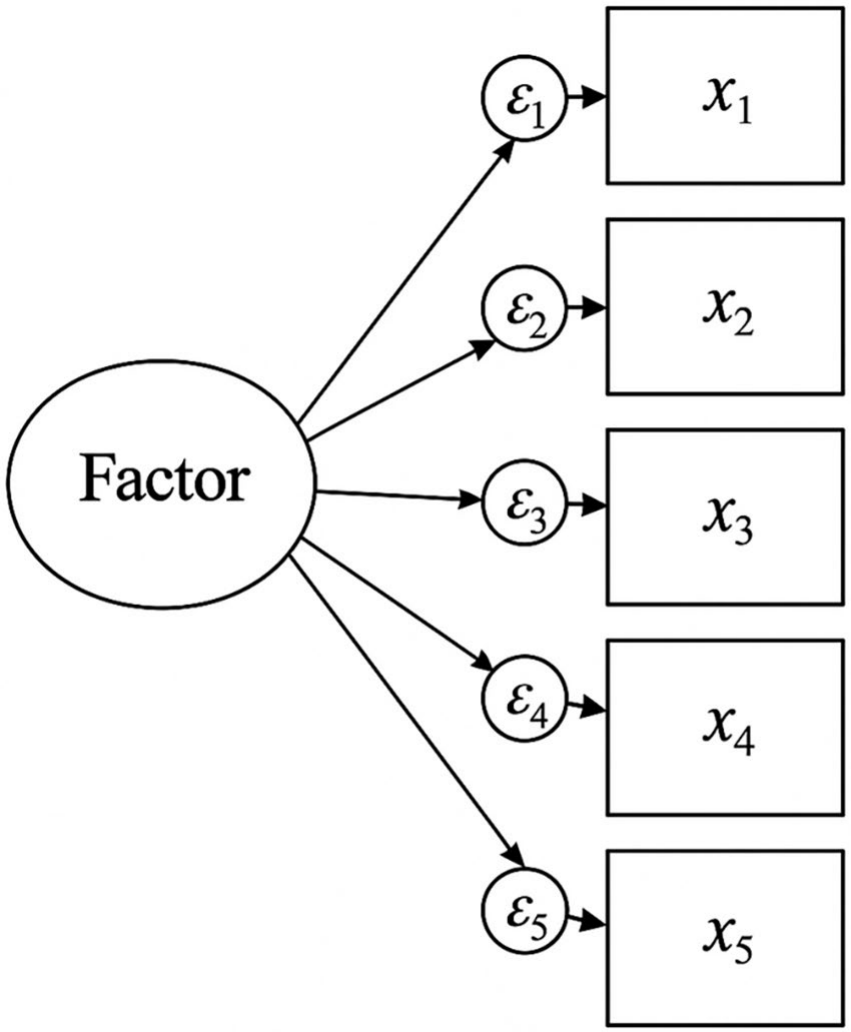
Path diagram of confirmatory factor analysis.

There were also 10 medical staff members, denoted as N1-N10. Their ages ranged from 28 to 46 years old, with an average age of 37 years (SD = 6.1). There were 5 male and 5 female staff members, and 5 had undergraduate degrees or above (Figure 1). The detailed information is shown in Table 2.

**Table 2:** General information of medical staff (*n* = 10).

Serial number	Gender	Age	Education background	Professional title	Years of work	Work position
N1	Male	43	Master’s Degree	Chief Physician	17	Doctor
N2	Female	32	Bachelor’s Degree	Nurse-in-Charge	13	Nurse
N3	Female	28	Bachelor’s Degree	Nurse Practitioner	6	Nurse
N4	Male	37	Master’s Degree	Attending Physician	12	Doctor
N5	Male	40	Master’s Degree	Associate Chief Physician	15	Doctor
N6	Female	33	Bachelor’s Degree	Nurse-in-Charge	14	Nurse
N7	Male	46	Doctoral Degree	Chief Physician	15	Doctor
N8	Female	45	Bachelor’s Degree	Associate Head Nurse	26	Head Nurse
N9	Female	33	Bachelor’s Degree	Nurse-in-Charge	11	Doctor
N10	Male	34	Doctoral Degree	Associate Chief Physician	7	Doctor

Content validity refers to the appropriateness and correspondence of the scale items in reflecting the measured content. The Likert 5-point scale was used to ask experts to rate the relevant content of each item in the scale. The Item-Content Validity Index (I-CVI) is calculated as the number of experts who gave a score of 4 or 5 divided by the total number of experts. Based on the total number of experts who gave a score of 4 or 5 for each item in the scale divided by the total number of consulting experts, the I-CVI of the scale was calculated to be 0.878–1.000, and the Scale-Content Validity Index (S-CVI) was 0.942 (Table 3).

**Table 3:** Key results from qualitative interviews and Delphi consultations.

Panel A: Main themes from qualitative interviews (*n* = 22).		
Dimension	Main themes	Number of mentions
Daily Care Needs	Mobility assistance, Shopping support, Environmental modifications, Diet planning, Fall prevention	5–6 per theme
Medical Nursing Needs	Rehabilitation training, Skin care, Urination function recovery, Catheter care	4–6 per theme
Health Education Needs	Product usage, Lifestyle modification, Medication knowledge	3–5 per theme
Social Participation Needs	Learning activities, Social engagement	3–4 per theme
Psychological Comfort Needs	Family support, Professional respect, Psychological counseling, Peer communication	4–7 per theme

B Expert consultation results (*n* = 22 experts).				
Round	Response rate	Mean importance score	Coefficient of variation	Items retained
Round 1	100% (22/22)	3.14–4.91	0.059–0.309	44/48 items
Round 2	100% (22/22)	4.18–4.96	0.021–0.213	36/36 items

Four items were deleted after Round 1 due to CV > 0.25. The naming of dimensions was based on consensus among the expert panel, with names chosen to reflect the content of items within each dimension and to align with the Z-R model framework.

The scale demonstrated good internal consistency reliability. The total scale and all dimensions showed acceptable to excellent Cronbach’s *α* values, indicating reliable measurement of the constructs. Similarly, the split-half reliability values confirmed the internal consistency of the scale (Table 4).

**Table 4:** Participant characteristics for scale validation (*n* = 530).

Characteristic	Exploratory FA sample (*n* = 250)	Confirmatory FA sample (*n* = 280)
Gender		
Male	112 (44.8%)	126 (45.0%)
Female	138 (55.2%)	154 (55.0%)
Age		
60–80 years	176 (70.4%)	195 (69.6%)
≥80 years	74 (29.6%)	85 (30.4%)
Type of UI		
Stress	128 (51.2%)	141 (50.4%)
Urge	7 (2.8%)	9 (3.2%)
Mixed	15 (6.0%)	18 (6.4%)
Overflow	100 (40.0%)	112 (40.0%)
Nocturia Episodes		
0–1 times/night	31 (12.4%)	35 (12.5%)
2–3 times/night	166 (66.4%)	183 (65.4%)
>3 times/night	53 (21.2%)	62 (22.1%)

For test–retest reliability, 50 participants completed the scale again after a two-week interval. The intraclass correlation coefficient (ICC) for the total scale was 0.847 (95% CI: 0.779–0.915), and the ICCs for each dimension ranged from 0.783 to 0.892, indicating good stability of the scale over time (Table 5).

**Table 5:** Factor analysis and reliability results.

Panel A: Exploratory factor analysis results (*n* = 250).
KMO = 0.948, Bartlett’s test: χ^2^ = 7671.663, *p* < 0.001.
Total variance explained: 67.643%.
Factor loadings range: 0.465–0.927.
One item deleted due to low loading (<0.4).

Panel B: Confirmatory factor analysis model fit (*n* = 280).			
Index	Value	Reference Value	Interpretation
χ^2^/df	1.412	≤3	Excellent
RMSEA	0.037	≤0.08	Excellent
SRMR	0.042	≤0.08	Excellent
GFI	0.901	>0.9	Good
TLI	0.942	>0.9	Good
CFI	0.947	>0.9	Good
NFI	0.915	>0.9	Good
IFI	0.948	>0.9	Good

Panel C: Reliability indices.				
Dimension	Items	Cronbach’s α	Split-half	Test–retest ICC
Daily Care Needs	13	0.893	0.828	0.892
Medical Nursing Needs	6	0.827	0.809	0.845
Health Education Needs	9	0.861	0.896	0.871
Social Participation Needs	2	0.757	0.712	0.783
Psychological Comfort Needs	5	0.772	0.701	0.812
Total Scale	35	0.901	0.865	0.847

To evaluate criterion-related validity, the correlation between our scale and the Incontinence Quality of Life (I-QOL) instrument was analyzed. The Pearson correlation coefficient between the total scores was −0.675 (*p* < 0.001), indicating a moderate negative correlation. This suggests that higher nursing needs are associated with lower quality of life, which aligns with clinical expectations and supports the criterion-related validity of our scale (Table 6).

**Table 6:** Validity assessment results.

Validity type	Index	Value	Interpretation
Content validity	I-CVI	0.878–1.000	Excellent
	S-CVI	0.942	Excellent
Criterion validity	Correlation with I-QOL	r = −0.675**	Moderate negative correlation
Discriminant validity	AVE square root	0.753–0.930	All > inter-factor correlations

**p < 0.001.

The Nursing Needs Scale for Elderly Patients with Urinary Incontinence (Formal Version) mainly consists of 5 dimensions, including 13 items for daily care needs, 6 items for medical nursing needs, 9 items for health education needs, 2 items for social participation needs, and 5 items for psychological comfort needs, with a total of 35 items. Each item has 5 options: “not needed,” “not too needed,” “somewhat needed,” “needed,” and “very needed,” which are scored from 1 to 5 points in sequence. The total score of the questionnaire ranges from 35 to 175 points, and a higher score indicates a higher degree of nursing care needs.

Clinical Interpretation Guide: To enhance the practical utility of the scale, we have developed the following score interpretation guidelines:

Low level of needs (35–70 points): Indicates minimal nursing care requirements, suitable for patients with mild urinary incontinence who maintain good independence.

Moderate level of needs (71–140 points): Indicates average nursing care requirements, appropriate for patients with moderate symptoms who need regular assistance.

High level of needs (141–175 points): Indicates intensive nursing care requirements, suitable for patients with severe urinary incontinence who need comprehensive care.

These interpretations should guide clinicians in developing appropriate care plans based on the assessed needs.

## 8 Discussion

This study successfully developed a nursing needs scale for elderly patients with Urinary Incontinence through a series of rigorous procedures, including literature analysis, qualitative interviews, the Delphi method, pre-testing, and questionnaire surveys. The scale addresses a critical gap in the Chinese healthcare system where existing assessment tools fail to capture the unique needs of elderly UI patients within their cultural and healthcare context. By integrating relevant literature and the actual experiences of patients and medical staff, the scale covers five dimensions: daily care needs, medical nursing needs, health education needs, social participation needs, and psychological comfort needs. This multi-dimensional design can more comprehensively reflect the complex needs of elderly patients with urinary incontinence.

The content validity of the scale was ensured through expert consultations. The two-round Delphi method involved 22 experts with rich experience and expertise in relevant fields. The high response rate and authority coefficient of the experts indicate their active participation and high-level professional judgment. The scale’s content validity was confirmed with excellent I-CVI and S-CVI values, suggesting effective measurement of nursing needs.

The scale demonstrated strong psychometric properties across multiple reliability indicators. This means that the items within each dimension are closely related and can stably measure the corresponding aspects of nursing needs. The test–retest reliability provides additional evidence of the scale’s temporal stability, indicating consistent results when administered to the same individuals at different time points.

The structural validity of the scale was evaluated through exploratory factor analysis and confirmatory factor analysis (24). The excellent KMO value and significant Bartlett’s test indicated the data’s suitability for factor analysis. The five-factor structure explained a substantial portion of variance, exceeding recommended thresholds for social science research.

However, some items showed cross-loadings between factors, suggesting potential areas for refinement. Future iterations of the scale should consider revising items with ambiguous factor loadings to improve discriminant validity. Specific strategies include: (1) rewording items to more clearly align with their intended dimension; (2) conducting cognitive interviews with patients to understand how they interpret potentially ambiguous items; (3) removing items that consistently load on multiple factors; and (4) developing new items through additional qualitative research to replace problematic ones.

The confirmatory factor analysis results supported the five-factor model with all fit indices meeting or exceeding recommended values. This indicates that the scale has a stable factor structure and can accurately measure the latent variables of nursing needs in elderly patients with urinary incontinence (25).

The criterion-related validity analysis showed a moderate negative correlation between our scale and the I-QOL instrument. This finding aligns with the logical expectation that higher nursing needs would be associated with lower quality of life in patients with urinary incontinence. This provides external validation of our scale and suggests it can effectively identify patients whose quality of life is impacted by their condition.

The developed scale has important clinical application value. It can provide a quantitative assessment tool for geriatric nursing practitioners, enabling them to more accurately evaluate the nursing needs of elderly patients with urinary incontinence. This is of great significance for formulating personalized care plans. For example, by understanding the specific needs of patients in daily care, medical nursing, and psychological comfort, nurses can provide targeted care measures, such as assisting patients with movement, providing skin care, and offering psychological support, which can effectively improve the quality of care (26).

The scale is particularly relevant in the Chinese healthcare context, where there are significant disparities between urban and rural areas in terms of access to specialized care and incontinence products. In urban settings, the scale can help healthcare providers optimize resource allocation and ensure comprehensive care delivery. In rural areas, where professional nursing resources are limited, the scale can guide family caregivers in identifying priority areas for support and seeking appropriate professional help when needed. The inclusion of items related to family support and traditional care practices reflects the important role of family in elderly care within Chinese culture.

Beyond the pandemic-related recruitment challenges, other confounding factors may have influenced participants’ responses. The pandemic context likely heightened social isolation, potentially amplifying psychological needs while reducing opportunities for social participation. Healthcare system strain during this period may have affected participants’ perceptions of medical and nursing needs. Future validation studies should reassess the scale in post-pandemic conditions to ensure its continued relevance and to identify any pandemic-specific response patterns that may need adjustment.

In addition, the scale can also be used to monitor the changes in patients’ nursing needs during the treatment and rehabilitation process. By regularly assessing patients’ needs, medical staff can adjust the care plan in a timely manner to ensure that the care provided is consistent with the patients’ actual situation. This can not only enhance the effectiveness of care but also promote the recovery of patients. Moreover, the scale can contribute to the standardization of nursing practice for elderly patients with urinary incontinence. It provides a unified assessment standard, which is conducive to the communication and cooperation among different medical staff and different medical institutions. This helps in the accumulation of clinical data and the improvement of the overall quality of nursing care for this patient population.

The developed clinical interpretation guide provides a framework for translating scale scores into meaningful categories of care needs. This guide will help healthcare providers to not only assess the level of needs but also to develop appropriate care plans based on the assessment results. For instance, patients with high scores (141–175) would benefit from intensive nursing interventions including frequent physical assistance, regular skin assessments, comprehensive health education, organized social activities, and scheduled psychological counseling.

Compared with existing general assessment tools for the elderly, such as those from Japan and Germany, the scale developed in this study has better specificity for elderly patients with urinary incontinence. General assessment tools may not fully address the unique needs of this specific group, such as the specific requirements for urinary tract infection prevention (27), bladder function rehabilitation (11), and psychological support related to urinary incontinence (28). The scale developed in this study takes these unique needs into account, making it more targeted and practical for clinical use.

Despite the good reliability and validity of the scale, this study has some limitations. First, due to the influence of time, manpower, and the COVID-19 pandemic, the sample in this study was only selected from two tertiary-level hospitals in Shanghai. The sample size and regional scope are limited, which may affect the generalizability of the scale. The relatively young mean age of participants (67 years) compared to the general UI population, the underrepresentation of OAB patients, and the high proportion of males with overflow incontinence suggest potential selection bias. These factors may have been influenced by pandemic-related restrictions on hospital access and patient recruitment. Future research could expand the sample size and include samples from different regions, such as community-dwelling elderly, nursing home residents, and patients from different levels of hospitals across the country. This would help to further verify the effectiveness and applicability of the scale in different populations and settings (4).

Second, although we conducted criterion-related validity testing against the I-QOL instrument, it would be valuable to compare our scale with other established nursing assessment tools specifically designed for elderly patients or those with urinary incontinence. This would provide a more comprehensive understanding of how our scale performs in relation to existing tools and could identify any unique contributions or potential redundancies.

Third, the factor analysis revealed some items with cross-loadings between factors, suggesting that the discriminant validity of these items could be improved. Future refinements of the scale should consider revising or replacing these items to achieve clearer factor structure and better measurement precision.

Fourth, our clinical interpretation guide needs further validation to ensure that the proposed score ranges accurately reflect meaningful differences in care needs. Longitudinal studies examining how scores relate to actual care requirements and outcomes would strengthen the clinical utility of the scale.

Fifth, while our study included clinical psychologists in the expert panel during scale development, the absence of psychologists in the qualitative interview phase represents a limitation. Given that psychological comfort needs emerged as a distinct dimension, direct input from mental health professionals during the interview phase could have provided deeper insights into the psychological aspects of UI. Future studies should include multidisciplinary perspectives from the outset, incorporating psychologists, physiotherapists, and social workers in all phases of scale development.

Finally, the scale has not been applied in clinical practice in depth. Future studies can use this scale to conduct surveys in actual clinical settings, observe the changes in patients’ conditions and needs over time, and explore the impact of different nursing interventions based on the scale assessment results. This will help to further optimize the scale and provide more practical guidance for clinical nursing.

## 9 Conclusion

This study successfully developed a nursing needs scale for elderly patients with urinary incontinence through a systematic methodology. The 35-item scale across five dimensions demonstrated strong psychometric properties and addresses a critical gap in assessment tools for this population within the Chinese healthcare context. The scale provides healthcare professionals with a culturally appropriate and clinically relevant tool for comprehensive needs assessment. While limitations exist, including pandemic-related recruitment challenges and the need for broader multidisciplinary input, the scale represents a significant advancement in standardizing care for elderly UI patients. Future research should focus on validating the scale in diverse populations and settings, refining items with cross-loadings, and establishing its predictive validity for care outcomes.

## Data Availability

The original contributions presented in the study are included in the article/supplementary material, further inquiries can be directed to the corresponding author.
